# Development and validation of quantitative thin layer chromatographic technique for determination of total aflatoxins in poultry feed and food grains without sample clean-up

**DOI:** 10.5455/javar.2021.h558

**Published:** 2021-11-06

**Authors:** Bahauddeen Salisu, Siti Marwanis Anua, Wan Rosli Wan Ishak, Nurzafirah Mazlan

**Affiliations:** 1 Department of Microbiology, Umaru Musa Yaradua University Katsina, Katsina, Nigeria; 2 School of Health Sciences, Health Campus Universiti Sains Malaysia, 16150 Kubang Kerian, Kelantan, Malaysia; 3 Department of Diagnostic and Allied Health Sciences, Faculty of Health and Life Sciences, Management and Science University, Selangor, Malaysia

**Keywords:** *Aflatoxins*, *foodgrains*, *poultry feed*, *limit of detection*, *preparative TLC*, *validation*

## Abstract

**Objective:**

The purpose of this work is to develop and validate an appropriate solvent solution and quantitative thin layer chromatography (TLC) method for determining the aflatoxins content of chicken feeds and dietary grains.

**Materials and Methods:**

To obtain the optimal mobile phase, samples were extracted with methanol/water (3:1) + 5% sodium chloride and partitioned using several solvent systems using preparative TLC. Camag TLC scanner 3 was used to scan the TLC plates at 366 nm and quantify them using JustTLC software. The method was tested for linearity, specificity, accuracy, precision, sensitivity, and robustness in accordance with ICH recommendations, and then utilized to screen 132 Nigerian poultry/food samples for total aflatoxins (TAFs).

**Results:**

The best separation of aflatoxins was achieved using acetonitrile and dichloromethane (3:17) mobile phase over an average run time of 45 min, resulting in linear calibration curves (*R^2^* > 0.99) in the concentration range limit of quantitation (LoQ) to 50 ng/spot with a limit of detection of <2.0 ng/g and a LoQ of <4.0 ng/gm for all aflatoxins in all spiked samples. When the proposed TLC method was compared to an optimized high-performance liquid chromatography method, an excellent linear regression was obtained (*R^2^* > 95%). Seventy seven (58.33%) of the 132 samples examined were positive for aflatoxins, with mean values ranging from 3.57 ± 2.55 to 37.31 ± 34.06 ng/gm for aflatoxin B1 and 6.67 ± 0.00 to 38.02 ± 31.52 ng/gm for TAFs, respectively.

**Conclusions:**

The results demonstrate the feasibility of using the suggested TLC method in conjunction with a novel solvent solution (free of carcinogenic chloroform) for the rapid and accurate measurement of TAFs in foods/feeds.

## Introduction

Numerous mycotoxigenic fungus, most notably *Aspergillus* species (spp)., *Penicillium* spp., and *Fusarium* spp., can infect numerous agricultural products both during and after harvesting, releasing toxic secondary metabolites known as mycotoxins that are extremely dangerous to humans, plants, and animals. Although the majority of foods are screened for the presence of mycotoxins prior to being released to consumers, research has shown that the majority of fungal spores germinate into fungi, and thus accumulate more mycotoxin in stored products under favorable environmental conditions [1,2], resulting in severe acute or chronic manifestations of mycotoxicoses in humans or other animals that consume such products. Thus, the development of simple, rapid, and reliable approaches for mycotoxin screening in foods and feeds is critical.

Section on *Aspergillus* spp., Flavi are the most commercially significant mycotoxigenic fungus at the moment, frequently contaminating poultry feeds [3–5] and stored food goods [6–12]. They create the most toxic/carcinogenic category of mycotoxins known as aflatoxins, of which two aflatoxin B groups [aflatoxin B1 (AFB1) and aflatoxin B2 (AFB2)] and two aflatoxin G groups [aflatoxin G1 (AFG1) and aflatoxin G2 (AFG2)] are the most hazardous and frequently encountered in foods and feeds [13–18]. These four aflatoxins are responsible for the majority of acute and chronic forms of aflatoxicosis in vertebrates worldwide. As a result, the critical nature of continual monitoring for the presence of these lethal poisons in meals and feeds cannot be overstated.

Thin-layer chromatography (TLC) continues to be one of the most straightforward, quick, and reliable procedures for screening aflatoxins in foods and feeds. It is 100% as effective as the enzyme-linked immunosorbent assay approach for assessing aflatoxins in fungal cultures [19]. It has been utilized in a number of recent investigations to detect and quantify aflatoxins in foods [20–30], feeds [31–35], and a variety of different matrices [19,20,36–47]. However, concerns with extraction methods and solvent systems remain unresolved. As can be seen from the majority of the above-cited literature, chloroform/acetone (9:1 v/v) is one of the most often used Association of Official Analytical Chemists approved solvent systems because it provides excellent TLC separation of aflatoxins. However, due to chloroform’s established oral and respiratory carcinogenicity and hepatotoxicity [48,49], its use is discouraged. The majority of known TLC solvent solutions for aflatoxins screening in foods or feeds are restricted to a single food or feed category. Thus, the objectives of this study are to (1) develop and validate a solvent system/TLC method for the detection and quantification of aflatoxins in foods and feed using a simple extraction process that does not require sample cleaning, (2) compare the method’s performance to that of a gold standard (STD) method [high-performance liquid chromatography (HPLC) method], and (3) assess the developed TLC method’s versatility by applying it to the screening of total aflatoxins (TAFs) in commercial poultry feeds, peanuts, and cereal samples from Nigerian open markets. The proposed technique can be used for both qualitative and quantitative aflatoxins determinations in feeds and foods with high precision and reliability; thus, it has the potential to become a routine technique for monitoring foods and feeds, particularly in laboratories lacking HPLC/high-performance thin layer chromatography/liquid chromatography with tandem mass spectrometry.

## Materials and Methods

### Reagents and STDs

The methanol (MeOH) used to extract the samples was of analytical grade obtained from R & M chemicals (United Kingdom). Other solvents used for the mobile phase selection were all HPLC - grade solvents with percentage purities greater than 99.9%. They include acetonitrile (ACN) [A998-4, Fisher Scientific, United State of America (USA)], acetone (A949-1, Fisher Scientific, USA), MeOH (106007, Merck KGaA, Darmstadt, Germany), dichloromethane (DCM) (106044, Merck KGaA, Darmstadt, Germany), acetic acid (02002123, Fisher Scientific, USA), chloroform (102445, Merck KGaA, Darmstadt, Germany), and toluene (108327, Merck KGaA, Darmstadt, Germany). The mixed aflatoxin STD (Pribolab, STD#1089) at a certified concentration of 20 μg/ml for each of the four aflatoxins (B1, B2, G1, and G2) in 5 ml ACN solution was supplied by Pribolab, Singapore.

### Food samples and poultry feed samples

For TAF determination, 132 composite samples of commercial poultry feeds and food grains were purchased from open markets in Nigeria (Katsina state). The samples purchased include 84 food grain samples comprising 21 composite samples each of maize, peanuts, wheat, and rice; 48 commercial poultry feed samples consisting of 12 composite samples each of starter, layer, grower, and finisher feeds. Each composite sample was made by combining three samples of the same type and brand purchased from different vendors of the same market.

### Preparation of fortified samples

A 100 ml working STD solution containing 200 ng/ml of AFB1, AFB2, AFG1, and AFG2 was prepared by diluting 1.0 ml of the 20.0 μg/ml mixed STD aflatoxin solution in 99 ml of MeOH. The working STD was used for spiking the food grains and poultry feed at various desired spiking levels. Premium quality poultry feed, peanut, maize, rice, and wheat samples were purchased for the spiking. The samples were screened to ensure they had no detectable levels of aflatoxins by HPLC. For each spiking, the sample was divided into two portions (spiking portion and blank), weighing 5 gm each. The blank portion was spiked with MeOH only. While the spiking portion was fortified with the desired spiking concentration using the spiking formula [50,51], below:


$$\mathrm{Aflatoxin}\;\mathrm{STD}\;\mathrm{to}\;\mathrm{spike}\;(\mathrm{ml})=\frac{\mathrm{Sample}\;\mathrm{weight}\;(\mathrm{gm})\times \mathrm{Desired}\;\mathrm{spiking}\;\mathrm{level}\;(\mathrm{ng}/\mathrm{gm})}{\mathrm{Concentration}\;\mathrm{of}\;\mathrm{aflatoxin}\;\mathrm{STD}\;(\mathrm{ng}/\mathrm{ml})}$$

The spiked samples were homogenized adequately to ensure even distribution of the aflatoxin, then equilibrated at room temperature for 1 h, and subsequently grounded to crystalline powder using an electric powder grinder (Golden Bull Stainless Steel Universal Miller, Bosch Teknologi, Malaysia). The grounded power was extracted based on the extraction method for the samples as described below.

### Extraction of aflatoxins from the samples

The samples were extracted by utilizing the double extraction protocol of aflatoxins [52] coupled with the principle of the quick, easy, cheap, effective, rugged, and safe extraction technique [53,54] with some modifications. Four different solvents, including MeOH + water (75 + 25 v/v), MeOH (100%), ACN + water (75 + 25 v/v), and ACN (100%), plus sodium chloride (NaCl) (5%), each was screened for aflatoxin extraction efficiency using poultry feed, rice, maize, wheat, and peanut samples spiked with aflatoxins at three levels (10, 20, and 30 ng/gm). In each case, 5 gm of the grounded spiked sample was weighed into a separate conical flask and extracted with 50 ml of the solvent/salt mixture by percolation for 2 h with continuous shaking at 100 rpm on an orbital shaker (Scilogex LCD digital orbital shaker, Westwood USA). The mixture was subsequently blended for 2 min using a high-speed blender, allowed to settle for 1 h before decanting and filtering the organic layer through filter paper (Smith 102 Qualitative A0335). The residue was remixed three more times with 20 ml of the extraction solvent, and each time allowed to settle for 1 hour, then decanted and filtered the organic layer. The total filtrate for each sample was evaporated in an evaporating oven at 300C, after which the resulting extracts were resuspended in 2 ml of the extraction solvent (without salt) and analyzed by HPLC. The solvent that gave the highest recovery of the aflatoxins was chosen and used throughout the rest of the study.

### Quantification of aflatoxins using HPLC

The HPLC analysis was carried out following the described protocol in the enclosed reference material certificate of analysis of the aflatoxin STDs (solutions in mycotoxin testing, Pribolab, Singapore) with minor modifications to achieve optimum separation of the aflatoxins. The analysis was carried out using HPLC Shimadzu, Nexera-LC-20AD (Shimadzu, Japan). Optimal chromatographic condition for the separation of all the four aflatoxins was achieved using a mobile phase consisting of MeOH + water (37:53) at 360 nm (excitation wavelength), 450 nm (emission wavelength), a flow rate of 0.50 ml/min, and an injection volume of 10.0 μl, in an Agilent HC-C18 (2) 150 × 4.60 mm 5.0 μm column (Agilent Technologies, Netherland). The total run time was 30 min at a column oven temperature of 400C.

A set of five different dilutions (1.0, 2.0, 4.0, 6.0, and 8.0 ng/ml) of the aflatoxin STDs were prepared from the stock solution of the aflatoxin mixture and analyzed by the HPLC at replicates (*n* = 3), after which a calibration curve of the average peak area versus the concentration of each aflatoxin was constructed using the post-run HPLC software (UFLC Shimadzu, Japan). The aflatoxin STDs’ calibration curve was used to assess the aflatoxins’ recovery in all the spiked samples.

### Screening of solvent systems for the qualitative detection of TAFs by preparative TLC (pTLC)

Fifty silica gel coated aluminum sheets, measuring 2.5 ×20 cm each, were cut from commercial aluminum TLC plates precoated with silica gel of 0.2 mm thickness (Merck 60 *F*_254_, Germany). A sample spotting point was made with a thin horizontal line drawn with a pencil from 2 cm to the bottom of each TLC sheet. To obtain a spotting amount of 20 ng/spot for each aflatoxin, 10 μl of the 200 ng/ml mixed STD aflatoxin solution was spotted at the middle of the spotting point of each TLC sheet and allowed to stay for 15 min at room temperature for the solvent to evaporate. Various solvent systems (mobile phases) composed of ACN/MeOH/H_2_O, ACN/H_2_O, ACN/acetone, ACN/DCM, acetone/DCM, MeOH/DCM, MeOH/H_2_O, and toluene/DCM at various ratios were tested for the ability to separate the aflatoxins on the TLC plates. Each TLC sheet was developed separately in the TLC tank using a single solvent system (mobile phase). The TLC plate for each solvent system was viewed using both ultra violet (U.V.) transilluminator at 365 nm and Camag TLC visualizer at 366 nm to observe the separation, if any, of the aflatoxins based on their characteristic fluorescence under UV light. Mobile phases that produced the excellent separation of the aflatoxins were selected and further screened for the ability to separate the four aflatoxins in spiked samples of peanut, maize, rice, wheat, and poultry feed spotted on the same TLC plate alongside the mixed aflatoxin STD at a distance of 2.5 cm parallel to each other. The best solvent system that produced the best separation of the aflatoxins in all the spiked samples was selected for the quantitative TLC and validation.

### Quantitative thin layer chromatography (qTLC) analysis

Prior to qTLC, aflatoxin bands obtained from the selected mobile phase in pTLC were carefully scraped, dissolved in MeOH, filtered using a microsyringe filter of 0.22 μm pore size (Microlab Scientific, Malaysia), and analyzed separately by HPLC to confirm the identity of the aflatoxin in each TLC band. The qTLC analysis was performed on 20 × 20 cm commercially developed aluminum TLC plates coated with silica gel of 0.2 mm thickness (Merck 60 *F*_254_, Darmstadt, Germany). A horizontal line was made with a pencil at 2 cm from the bottom of each plate and 1 cm from the top to serve as a mark for sample application point and solvent font. Samples were spotted (10 μl per spot) using a micropipette at 2.5 cm from one spot to another on the plates along the sample application line. The plates were pre-washed with the mobile phase before the sample application. Spotted plates were developed separately in the TLC tank (Kontes, Vineland, USA) containing 200 ml of the mobile phase, after which the plates were dried for 1 h at room temperature. The developed TLC plates were scanned at 366 nm with Camag TLC scanner 3, and the aflatoxin bands were quantified using JustTLC software (version 4.5 Sweday, Lund, Sweden).

### Validation of the qTLC method

The method was validated following the protocol of the ICH (International Council for Harmonisation of Technical Requirements for Pharmaceuticals for Human Use) [55] for linearity, specificity, accuracy, repeatability, reproducibility (intermediate precision), robustness, and sensitivity [limit of detection (LoD), and limit of quantitation (LoQ)].

## Linearity

The method’s linearity was determined by spotting ten different concentrations (0.25, 0.5, 1.0, 2.0, 4.0, 8.0, 16.0, 32.0, 64.0, and 100.0 ng/spot) of the aflatoxin solution on the 20 × 20 cm TLC plate and developed in TLC tank using the selected mobile phase, after which the plate was scanned at 366 nm with Camag TLC scanner 3 and processed using the JustTLC software to obtain the volume of the four aflatoxin bands for each concentration. The same procedure was repeated twice to obtain a triplicate set. A five-point calibration curve was constructed by plotting the average volume of aflatoxin bands against the concentration (ng/spot). The regression equation (for the relationship between the volume of aflatoxin bands and concentration) obtained for each of the four aflatoxins was used to determine its respective recovery in the spiked and unknown samples.

## Sensitivity (LoD and LoQ)

The method’s sensitivity was evaluated by assessing the LoD and LoQ for each aflatoxin (AFB1, AFB2, AFG1, and AFG2). The determination of both parameters (LoD and LoQ) was carried out based on the standard deviation (SD) of responses (σ) and the slope (S) of the calibration curves for both the aflatoxin STD and the spiked extracts using the following formulae [55].


$$\mathrm{LoD}=\frac{3\sigma }{S}\;\;\;(1)$$

$$\mathrm{LoQ}=\frac{10\sigma }{S}\;\;\;(2)$$

### Repeatability (intra-day precision) and reproducibility (inter-day precision)

The repeatability was evaluated by spiking blank samples at three different concentrations within the linearity range, spotted together (50, 40, and 30 ng/spot) on TLC plates in triplicate, and developed on the same day. The within-day precision was then evaluated based on the variation in the obtained aflatoxin concentrations for the triplicate analyses expressed in percentage relative SD (% RSD).

The within laboratory reproducibility (intermediate precision), on the other hand, was assessed by repeating the intra-day (*n* = 3) precision analysis for two more different days, making a total of nine replicates of each concentration for the three different days. The intermediate precision was expressed in terms of the % RSD of the average concentrations of aflatoxins of the 3 days for each of the three spiked concentrations of the aflatoxins.

## Specificity

The method’s specificity was evaluated by analyzing the STD aflatoxin solution and the spiked extracts for the presence or absence of interfering bands within the retention factor (Rf) range regions of the four aflatoxins at three different concentrations for replicates (*n* = 3).

## Accuracy

The method’s accuracy was evaluated based on the STD addition method [55–57]. Three different concentrations of the aflatoxins (prepared by spiking blank solution of maize extract) were spotted on the TLC plate at 50, 40, and 30 ng/spot in triplicate. The plates were developed, and the accuracy was calculated as the average percentage recovery of the aflatoxins from the three developed TLC plates at each concentration.

## Robustness

The method’s robustness was ascertained by varying the extraction time, solvent saturation level during development, and slight changes in the mobile phase composition.

## Performance evaluation

The TLC method’s performance was evaluated using 10 dilutions (1.0, 2.0, 4.0, 8.0, 10.0, 12.0, 14.0, 16.0, 18.0, and 20.0 ng/ml) of the mixed aflatoxin STDs analysed by both the TLC method and the HPLC method. The performance of the two methods was then compared using the fit regression model and Bland-Altman’s method of assessing the degree of agreement between two quantitative methods [58]

### Screening of aflatoxins in commercial poultry feeds and foodgrains

The validated qTLC method was used to screen the 132 commercial poultry feed samples and food samples for TAFs (AFB1, AFB2, AFG1, and AFG2). All the samples were extracted as described above. The extract of each sample was dissolved in 2 ml of the extraction solvent (MeOH/water, 3:1 v/v) and filtered with the 0.22 μm pore size syringe filter (Microlab Scientific, Malaysia) before spotting on the TLC plates alongside the mixed aflatoxin STD (positive control) and analyzed by the qTLC as described above.

**Figure 1.**
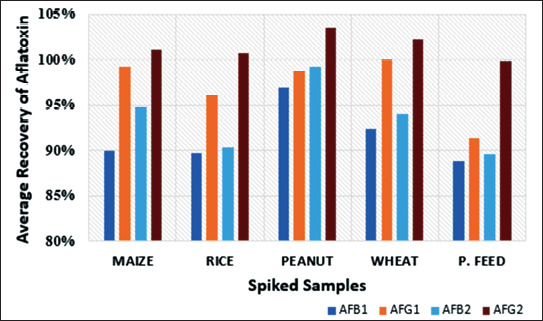
Average recoveries of the aflatoxins for three spiking levels in maize, rice, peanut, wheat, and poultry feed. Samples were extracted in triplicates using MeOH + water + NaCl (75:25:5, v/v/w).

## Results and Discussion

### Extraction of the aflatoxins

Like many other mycotoxins, aflatoxins have moderate polarity, making them readily soluble in mixtures of water and polar organic solvents such as ACN, acetone, or MeOH [59–64]. Hence, they are most commonly extracted by shaking in these solvents for 30–90 min and subsequently analyzed after filtering the extracts plus or minus a clean-up stage [64,65] also called masked mycotoxins. Mycotoxins are secondary fungal metabolites, toxic to human and animals. Toxigenic fungi often grow on edible plants, thus contaminating food and feed. Plants, as living organisms, can alter the chemical structure of mycotoxins as part of their defence against xenobiotics. The extractable conjugated or non-extractable bound mycotoxins formed remain present in the plant tissue but are currently neither routinely screened for in food nor regulated by legislation, thus they may be considered masked. Fusarium mycotoxins (deoxynivalenol, zearalenone, fumonisins, nivalenol, fusarenon-X, T-2 toxin, HT-2 toxin, fusaric acid. In the current protocol, a recovery study of aflatoxins from spiked maize, rice, wheat, peanut, and poultry feed was carried out using four different extraction solvents without applying the clean-up stage as described above. The average recovery values of aflatoxins obtained using the tested solvents at the three spiking levels include 42.37%–48.62%, 44.77%–47.08%, 79.11%–91.48%, and 88.81%–103.52% for 100% MeOH, 100% ACN, ACN + water (3:1 v/v), and MeOH + water (3:1 v/v) solvents respectively. Thus, lower aflatoxin recovery was obtained when 100% MeOH, 100% ACN, or ACN + water (3:1 v/v) plus salt were used as extraction solvent, which is in accordance with the reports of Choochuay et al. [54] and Capriotti et al. [66]. Hence, the extraction solvent consisting of a mixture of NaCl and MeOH + water (3:1 v/v) having the highest recovery/yield of aflatoxins in all the spiked samples was chosen for the present study. This chosen solvent has successfully extracted aflatoxins from the fungal culture [67] and poultry feeds [68]. Figure 1 shows the average recoveries of all the aflatoxins in all the spiked samples obtained using the chosen extraction solvent.

### Determination of aflatoxins using HPLC 

**Figure 2.**
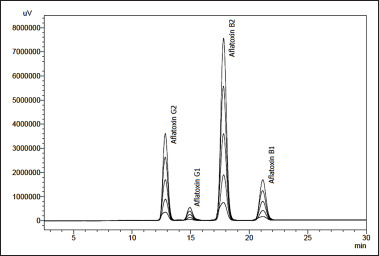
Chromatogram comparison showing the linearity in the HPLC analysis of five different concentrations (1.0, 2.0, 4.0, 6.0, and 8.0 ng/ml) of aflatoxin STDs prepared in 75% MeOH.

The linearity of the aflatoxin STDs obtained by HPLC is depicted in Figure 2. All the four aflatoxins were well separated at distinct retention times, producing sharp peaks with no interference of the sample matrix in all the spiked samples. The coefficient of determination (*R^2^*) values obtained from the linear calibration curves of the aflatoxins over the concentration range of 1.0 to 8.0 ng/ml are 0.9995, 0.9999, 0.9997, and 0.9993 for AFB1, B2, G1, and G2, respectively. The calibration curve for each aflatoxin was used to assess its level in all the spiked samples. The high *R^2^* values indicate the method’s high sensitivity [69,70], which was further ascertained by the high recovery values (99.6% to 107%) of aflatoxins in the spiked samples. Hence, the HPLC method was used to evaluate the performance of the qTLC method. 

### Screening of solvent systems for the qualitative detection of TAFs by pTLC

**Figure 3.**
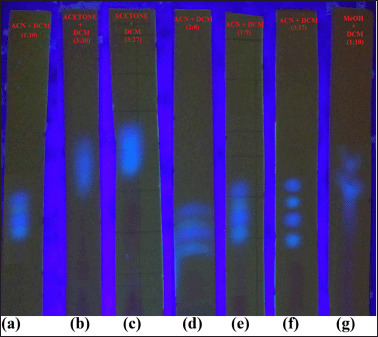
Pattern of aflatoxin separation produced by the solvent systems. Picture taken on UV transilluminator at 365 nm. (a) ACN + DCM (1:10, v/v), (b) Acetone + DCM (3:30, v/v), (c) Acetone DCM (3:27, v/v), (d) ACN + DCM (2:8, v/v), (e) ACN + DCM (1:9, v/v), (f) ACN + DCM (3:17, v/v), (g) MeOH + DCM (1:10, v/v).

The result of the preliminary screening of the various solvent systems for their ability to separate aflatoxins in the extracts of the spiked samples when used as TLC mobile phases are presented in Table 1. Of the 20 different solvent systems tested, only seven showed a considerable separation of the aflatoxins. In contrast, others only dragged the extract along with the TLC plates with little or no separation. Figure 3 shows the pattern of separation produced by the best seven solvent systems. As shown in Figure 3, all the seven suitable mobile phases contained DCM and ACN or MeOH. Since World War II, DCM has been a versatile organic solvent to dissolve different organic substances/compounds in many chemical and industrial processes [71]. It has more vital dielectric forces than chloroform, making it have higher polarity and elution power compared to the latter. 

**Figure 4.**
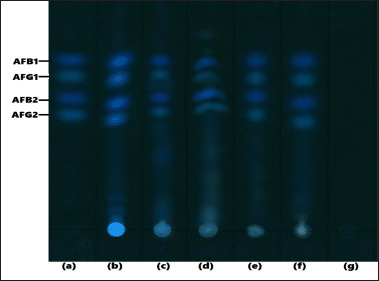
TLC Plate showing the pattern of aflatoxins separation in the extracts of the various spiked samples produced by ACN + DCM (3:17, v/v) mobile phase. The photo was taken by Camag TLC visualizer 3 at 366 nm. (a) Aflatoxin STD (positive control) (b) Spiked maize, (c) Spiked rice, (d) Spiked wheat, (e) Spiked peanut, (f) Spiked poultry feed, (gm) solvent (negative control).

Partitioning of compounds by TLC largely depends on the solubility of the compounds in the chosen mobile phase and their relative affinity to the stationary phase used [72]. Aflatoxins generally require fewer polar solvents for better separation in chromatographic conditions [52]; hence, their chromatographic separation often depends on the percentage of the organic solvents in the mobile phases [54,73,74]. Our result showed that the mobile phase consisting of ACN/DCM at a ratio of 3:17 v/v produced the best separation of all the four aflatoxins at distinct Rf values. Aflatoxins were not detected in mobile phases consisting of high polar solvents like water. A similar pattern was observed by [73] when they compared the performance of the highly polar and non-polar solvent system as mobile phases for separating aflatoxins. Therefore, the ACN-DCM 3:17 mobile phase was chosen and validated in this study. Figure 4 shows the level of separation produced by the selected mobile phase (ACN-DCM, 3:17, v/v) in all the spiked samples.

**Table 1.** TLC mobile phases screened for aflatoxin separation potential.

S/N	TLC mobile phase (solvent system) in ml ratios	Aflatoxins detected in each spiked sample				
		Poultry feed	Maize	Peanut	Rice	Wheat
1	ACN + MeOH + dH_2_O (20:20:60)	ND	ND	ND	ND	ND
2	ACN + MeOH + dH_2_O (10:30:60)	ND	ND	ND	ND	ND
3	ACN + MeOH (50:50)	ND	ND	ND	ND	ND
4	ACN + dH_2_O (16:84)	B1	B1	ND	B1	B1
5	ACN + Acetone (1:10)	ND	ND	ND	ND	ND
6	ACN + Acetone (1:9)	ND	ND	ND	ND	ND
7	ACN + DCM (3:20)	B1, B2	B1, B2	B1, B2	B1, B2	B1, B2
8	ACN + DCM (2:8)	TAFS	TAFS	TAFS	TAFS	TAFS
9	ACN + DCM (1:9)	TAFS	TAFS	TAFS	TAFS	TAFS
10	ACN + DCM (1:10)	TAFS	TAFS	TAFS	TAFS	TAFS
11	ACN + DCM (3:17)	TAFS	TAFS	TAFS	TAFS	TAFS
12	Acetone + DCM (1:10)	NAFS	NAFS	NAFS	NAFS	NAFS
13	Acetone + DCM (1:9)	NAFS	NAFS	NAFS	NAFS	NAFS
14	Acetone + DCM (1:5)	ND	B1	ND	B1	B1
15	Acetone + DCM (3:20)	ND	B1	ND	B1	B1
16	MeOH + DCM (1:4)	ND	ND	ND	ND	ND
17	MeOH + DCM (1:10)	NAFS	NAFS	NAFS	NAFS	NAFS
18	MeOH + DCM (1:9)	NAFS	NAFS	NAFS	NAFS	NAFS
19	MeOH + DCM (3:20)	ND	ND	ND	ND	ND
20	MeOH + dH^2^O + DCM (1:1:8)	ND	ND	ND	ND	ND

ACN = Acetonitrile, B1 = Aflatoxin B1, B2 = Aflatoxin B2, DCM = Dichloromethane, dH_2_O = Distilled water, MeOH = Methanol, NAFS = Not well-separated Aflatoxins, ND = Not detected, and TAFS = Total aflatoxins separation.

### Validation of the qTLC method: linearity, sensitivity, specificity, accuracy, precision, and robustness

**Figure 5.**
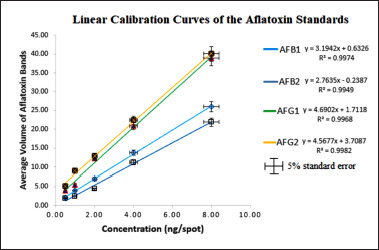
Linear calibration curves of the aflatoxin STDs. All the values were plotted with STD error of 5%.

The calibration curves for the aflatoxin STD solution and the spiked extracts showed excellent linearity with a coefficient of regression values (*R^2^*) above 99%. The linearity followed Beer’s law [75] over the concentration range of 0.5 to 8.0 ng/spot as ascertained by the high *R^2^* values and SD of intercept <5% and % RSD of <2%. Figure 5 showed the calibration curves of the four aflatoxins obtained by plotting the respective concentration of each aflatoxin against the average volume of TLC bands at replicates (n = 3) for each of the four aflatoxins. The overall sensitivity of the method as assessed by the LoD and LoQ values showed that the technique is highly sensitive with LoD of < 2.0 ng/gm and LoQ of < 4.5 ng/gm for all the aflatoxins in all the spiked samples. Table 2 shows the summary of the sensitivity test in spiked maize samples. 

The capacity of an analytical technique to differentiate the target analyte in the presence of additional components in the sample is referred to as specificity [55,76] This method was found to be specific as there was no interference of the aflatoxin bands with other components in the sample matrix in all the spiked samples. This shows that the method is highly selective.

**Table 2.** Sensitivity of the method in spiked maize samples.

Aflatoxins	Concentration (ng/spot)	Recovery (%)	% RSD	Linear regression curve			LoD (ng/g)	LoQ (ng/g)
				Equation	*R^2^*	SEI		
AFB1	0.5–8.0	81.06–96.99	0.31–1.62	*Y* = 2.4976x + 1.2688	0.9980	0.2686	0.81	2.46
AFB2	0.5–8.0	80.50–97.16	0.26–1.77	*Y* = 2.4455x + 0.6144	0.9977	0.2790	0.86	2.61
AFG1	0.5–8.0	75.40–95.03	0.20–1.70	*Y* = 4.0652x + 1.4833	0.9968	0.5529	1.03	3.11
AFG2	0.5–8.0	89.29–101.11	0. 35–1.68	*Y* = 4.5980x + 3.2991	0.9984	0.4449	0.73	2.22

where, SEI is the STD error of intercept.

The percentage recoveries of the aflatoxins are between 74% and 105% (Table 3), which is within the acceptable limit of 70%–10% set by the commission regulation of the European Communities [77] for aflatoxins measured at a concentration of 1 to 10 μg/kg. The method was also found to be both repeatable and reproducible, as ascertained by the lower percentage RSDs of < 2% for all the intra-day and inter-day precision measurements (Table 3). This shows that both the accuracy and precision of the method are outstanding.

### Method’s performance evaluation

**Figure 6.**
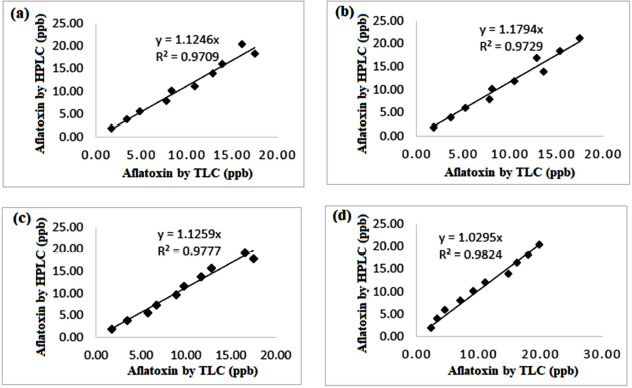
A regression plot showing the calibration of the aflatoxin concentration determined by the HPLC method and the proposed qTLC method for ten different concentrations (1.0 to 20.0 ng/ml) of (a) AFB1, (b) AFB2, (c) AFG1, and (d) AFG2.

A linear regression given in Figure 6 was used to compare the performance of the validated TLC technique to a gold STD method (HPLC method), yielding a good correlation (*R^2^* > 97%) between the two techniques [78]. The *Z*-reg equation’s slope aligns with the anticipated value that signifies a good agreement between the two techniques. 

**Figure 7.**
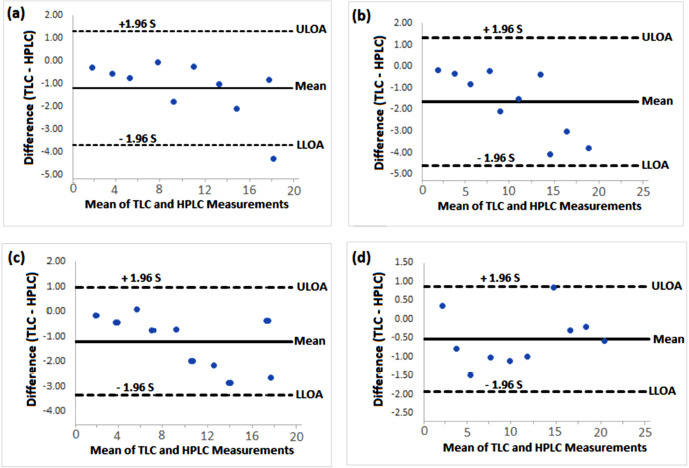
Bland and Altman plots showing the degree of agreement between the measurements of the various concentrations of aflatoxins (1. 0 to 20.0 ng/ml) by the TLC and HPLC methods for (a) AFB1, (b) AFB2, (c) AFG1, and (d) AFG2). S is the SD of the difference between the TLC and HPLC measurements, LLOA and ULOA are the respective lower and upper limits of agreements between the two methods.

In addition, when the level of agreement between the TLC method and the HPLC method was further assessed using Bland-Altman’s test, a good level of agreement between the two methods was obtained as evidenced by the smaller values of the mean difference (bias) between the two methods (Fig. 7). This implies that the TLC method could be used routinely to determine aflatoxins in both food grains accurately and poultry feeds with its simplicity and cost-effectiveness.

### Screening of aflatoxins in commercial poultry feeds and foodgrains

Of the 84 Nigerian food grains analyzed, 54 (64.29%) were contaminated by at least one or more aflatoxins at the concentration level ranging from 0.3 to 106.7 ng/gm (mean range = 3.61 ± 3.7 to 38.02 ± 31.52 (Table 4). Peanut samples have the highest aflatoxin contamination level, followed by maize samples, wheat, and rice samples. About 46% of the positive samples contained aflatoxins above the permissible limit of 20 μg/kg enforced in Nigeria and many countries such as the USA, Nepal, Austria, Philippines, Brazil, Kenya, etc. [79]. The mean and range of each aflatoxin in the positive samples are summarized in Table 4. AFB1 was present in 44 out of the 54 positive samples, including 8 samples contaminated by all the 4 aflatoxins, 18 samples contaminated by AFB1 only, and 18 other samples contaminated by AFB1 and 1 or 2 of the 3 other aflatoxins. This is followed, in decreasing order, by AB2, AG1, and AFG2, which were detected in 26, 25, and 15 of the 54 positive samples, respectively. However, AFB2 has the highest overall mean level, followed by AFB1, AFG2, then AFG1. The level of aflatoxins obtained from the food grains in this study were lower than the previously reported levels in some Nigerian foods such as peanut cake (mean = 151 to 1,428.4 μg/kg) [80], local snacks (range = 14 to 1,041 μg/kg) [81], stored maize (range = 26.5 to 15, 489.6 μg/kg) [82], dried tomatoes (range = 853 to 1,430 μg/kg) [83], peanuts (range = 0.0 to 2,076 μg/kg) [84], and cereals (mean range = 0.88 to 589.8 μg/kg) [85]. However, the level of aflatoxins in our study was higher than those reported by some other researchers from Nigeria [86–90].

**Table 3.** Summary of the method’s accuracy and precision analyses.

Sample categories	Aflatoxins analysed	Rf values		Precision at 50 ng/spot				Precision at 40 ng/spot				Precision at 30 ng/spot			
		Mean	SD	Within day n = 3		Between day n = 9		Within day n = 3		Between day n = 9		Within day n = 3		Between day n = 9	
				Recovery (%)	% RSD	Recovery (%)	% RSD	Recovery (%)	% RSD	Recovery (%)	% RSD	Recovery (%)	% RSD	Recovery (%)	% RSD
Blank extract + aflatoxin STD solution (accuracy)	AFB1	0.62	0.02	102.13	1.56	100.31	1.15	98.72	1.37	101.04	1.27	99.43	1.60	100.28	1.49
	AFB2	0.54	0.05	101.72	1.33	100.93	0.72	101.70	1.92	100.92	1.35	102.14	1.63	103.17	1.42
	AFG1	0.47	0.09	103.77	1.73	101.78	1.70	100.78	1.06	101.67	1.70	101.52	0.82	100.54	0.61
	AFG2	0.40	0.13	98.87	1.11	106.84	0.79	103.84	1.74	101.99	1.71	105.43	1.88	103.84	1.57
Spiked maize	AFB1	0.60	0.05	90.37	0.84	88.96	0.42	85.95	1.84	93.84	1.94	74.34	1.31	75.19	1.20
	AFB2	0.53	0.07	87.90	1.17	98.91	0.55	96.95	1.99	98.49	1.80	87.06	1.64	88.10	1.85
	AFG1	0.46	0.10	94.31	0.90	92.32	0.47	72.22	1.16	94.53	0.52	86.32	1.82	85.33	1.61
	AFG2	0.38	0.13	97.83	0.77	100.79	3.53	99.56	1.28	102.86	2.19	88.40	1.87	86.81	1.56
Spiked rice	AFB1	0.62	0.04	87.29	0.96	85.88	0.55	83.76	1.16	93.25	1.26	77.25	1.82	78.10	1.93
	AFB2	0.55	0.06	94.93	1.09	92.94	0.48	90.39	1.83	94.61	1.25	87.62	1.88	88.65	1.67
	AFG1	0.47	0.09	90.47	0.81	88.48	0.37	91.79	1.79	90.82	1.14	80.68	1.64	79.70	1.43
	AFG2	0.39	0.13	94.65	1.00	98.62	1.38	100.68	0.58	99.90	1.95	92.68	1.48	91.09	1.79
Spiked wheat	AFB1	0.59	0.12	92.22	0.77	100.80	1.84	99.55	0.62	95.41	0.52	82.82	0.29	83.67	0.18
	AFB2	0.51	0.14	86.92	1.10	101.93	1.87	96.01	1.46	87.50	0.89	86.56	0.45	87.59	0.24
	AFG1	0.44	0.17	84.54	0.82	97.55	0.38	102.55	1.06	98.17	1.54	85.62	0.41	84.85	0.19
	AFG2	0.38	0.21	88.81	1.81	102.77	0.99	103.23	1.72	85.63	2.08	93.31	0.58	91.72	0.27
Spiked peanut	AFB1	0.63	0.02	94.25	0.88	92.83	0.46	92.83	0.51	95.17	0.42	76.18	0.35	77.03	0.23
	AFB2	0.57	0.04	96.42	1.04	94.43	1.75	102.26	1.38	98.31	1.36	86.13	0.43	87.17	0.22
	AFG1	0.48	0.09	96.20	0.81	94.21	0.37	94.21	1.65	97.01	1.01	80.84	1.27	78.85	1.06
	AFG2	0.40	0.13	87.47	0.86	100.44	1.49	103.17	1.85	101.81	1.95	91.44	0.58	89.85	0.27
Spiked poultry feed	AFB1	0.61	0.11	89.49	1.98	88.08	0.46	88.08	1.57	89.82	1.48	75.69	1.50	76.55	1.39
	AFB2	0.56	0.13	93.91	1.09	91.92	0.48	91.92	1.39	93.29	1.96	75.71	0.45	76.74	0.24
	AFG1	0.47	0.16	91.70	0.89	89.71	0.46	89.71	1.11	92.71	1.76	75.13	0.44	74.15	0.23
	AFG2	0.41	0.19	96.88	1.89	100.84	1.35	102.51	0.53	99.61	1.80	87.04	0.68	85.45	0.37

Similarly, 23 (47.92%) of the 48 Nigerian poultry feed samples analyzed were contaminated by aflatoxins ranging from 1.02–103.82 ng/gm (mean range = 7.60 ± 5.34 to 82.34 ± 51.18 ng/gm) (Table 4), with about 45% containing the aflatoxins above the permissible limit of 20 μg/kg as set by the European Union (E.U) [79]. Unlike in the food grains, AFB1 has the highest detection frequency and mean level of contamination in the positive poultry feed samples, followed by AFB2, AFG2, and AFG1. The prevalence of aflatoxins in the poultry feed samples analyzed in the study was lower than those reported by other researchers in Nigeria [3,91–93]. However, the range of aflatoxin concentration in our samples was higher than those reported in some other parts of Nigeria [3,91,92] but lower than those reported by Momodu et al. [93].

The high level of aflatoxins obtained from the samples in this study calls for more preventive intervention/control strategies at various food/feed value chain levels to reduce the contamination level to the acceptable limits. Some of the significant factors that could be responsible for aflatoxin contamination of food products in Nigeria include:

**Table 4.** Level of aflatoxin contamination of food grains and poultry feed samples from Nigerian open markets.

Sample categories	*N*	AFB1		AFB2		AFG1				
		*N*	Concentration (ng/gm)	*N*	Concentration (ng/gm)	*N*	Concentration (ng/gm)		*N*	Concentration (ng/gm)
Maize	21	11	20.13 ± 10.47 (7.05–34.04)	2	24.34 ± 9.45 (17.66–31.02)	7	15.03 ± 12.57 (4.19–41.33)		2	16.93 ± 0.79 (16.37–17.49)
Peanut	21	10	24.19 ± 26.57 (1.49–66.83)	7	38.02 ± 31.52 (1.89–90.72)	5	36.07 ± 37.85 (0.31–91.03)		4	12.35 ± 9.09 (3.05–24.86)
Rice	21	9	9.65 ± 5.55 (0.97–19.05)	10	32.92 ± 19.57 (10.03–68.51)	3	9.07 ± 3.51 (6.17–13.09)		6	10.86 ± 6.22 (3.35–19.77)
Wheat	21	14	37.31 ± 34.06 (2.13–106.71)	7	34.23 ± 21.26 (3.35–57.14)	5	10.84 ± 8.59 (1.14–21.91)		3	15.17 ± 18.15 (2.41–35.95)
Poultry feed	48	20	82.34 ± 51.18 (20.51–103.82)	12	32.97 ± 14.96 (1.02–54.33)	4	27.60 ± 15.34 (3.27–35.21)		9	24.37 ± 9.68 (5.46–36.04)

Values for the concentration are mean ± SD, numbers in the brackets indicate the range of contamination from lowest to highest contamination levels, *n* = Total number of samples analysed, *N* = Number of positive samples for individual aflatoxin in the respective samples (it does not represent the overall total positive samples as some aflatoxins occurred in the same samples together).

1. Poor harvesting practices (e.g., most crops such as corn, millet, and peanuts are harvested during the rainy season and packed together as heap/stook in the fields and left until the dry season before they are collected for storage). The stook accumulates moisture and heat, which promote fungal proliferation and subsequent aflatoxin accumulation.
2. Most grain farmers and traders/sellers use poor storage facilities (e.g., leaky roofs and locked-up stores with insufficient ventilation outlets).
3. Inter transportation among stakeholders in open trucks under warm temperatures and high relative humidity.
4. Non-sorting of damaged/contaminated crops from uncontaminated ones during harvesting and storage.
5. Fluctuations in the relative humidity and temperatures in the open markets where the commodities are being sold and the general lack of knowledge of fungi and aflatoxins by most retailers lead to poor handling/hygienic practices of the commodities.

Thus, appropriate intervention strategies should address these issues to reduce the risks of aflatoxin exposure via contaminated foods or feeds.

## Conclusion

We have successfully developed and validated a new solvent system and TLC method for the simultaneous qualitative and quantitative determination of TAFs in foods and poultry feeds, following the ICH guidelines for validating an analytical method. The technique was found to be rapid, simple, coast effective, highly specific, reliable, accurate, repeatable, reproducible, robust, and sensitive with LoD and LoQ below 4 ng/gm for all the aflatoxins. Hence, the method could become a routine technique for determining TAFs in foods and feeds, especially in laboratories where HPLC/LCMS instruments are unavailable. Further research is recommended to investigate the applicability of the new solvent system for separation and detection of other mycotoxins in feeds and foods, as well as its application in aflatoxin partitioning in different matrices such as extracts from fungal cultures, where TLC is currently used as a barcode in aflatoxigenicity screening and differentiation between aflatoxigenic and non-aflatoxigenic *Aspergillus* spp. Some of the samples analyzed in this study contained aflatoxins above the maximum legal levels permitted by many countries and the E.U. Therefore, it is recommended that appropriate intervention/control strategies be implemented/strengthened at various food/feed value chain levels to reduce the high contamination rate of staple feeds and foods by the highly toxic/carcinogenic mycotoxins (aflatoxins) to acceptable levels.

## List of Abbreviations

<, Less than; >, Greater than; °C, Degree celsius; μg/ml, Microgram per milliliter; μl, Microliter; ACN, Acetonitrile; AFB1, Aflatoxin B1; AFB2, Aflatoxin B2; AFG1, Aflatoxin G1; AFG2, Aflatoxin G2; DCM, Dichloromethane; e.g., Example; E.U., European Union; gm, Gram; HPLC, High-performance liquid chromatography; ICH, International Council for Harmonisation; LLOA, Lower Limit of Agreement LoD, Limit of detection; LoQ, Limit of quantitation; MeOH, Methanol; NaCl, Sodium chloride; NAFS, Not well-separated aflatoxins; ND, Not detected; ng/gm, Nanogram per gram; ng/ml, Nanogram per milliliter; pTLC, Preparative thin layer chromatography; qTLC, Quantitative thin layer chromatography; *R*^2^, Coefficient of determination; Rf, Retention factor; RSD, Relative stsndard deviation; SEI, Standard Error of Intercept spp, Species; STD, Standard; TAFs, Total aflatoxins; TLC, Thin layer chromatography; UFLC, Ultra-Fast Liquid Chromatography; ULOA, Upper Limit of Agreement U.V., Utra violet; USA, United State of America; v/v, Volume by volume ratio.

## References

[1] Gamuchirai L, Muusha AB, Mashingaidze LM (2019). Effect of drying techniques and storage conditions on quality and incidence of aflatoxins in dried chillies (*Capsicum frutescens*) in Zimbabwe. Acta Sci Agric.

[2] Villers P, Kalule DO, Semi N (2014). Aflatoxins and safe storage. Front Microbiol.

[3] Kehinde MT, Oluwafemi F, Itoandon EE, Orji FA, Ajayi OI (2014). Fungal profile and aflatoxin contamination in poultry feeds sold in Abeokuta, Ogun State, Nigeria. Niger Food J.

[4] Ibrahim NM, Kabir J, Kwanashie C, Salawudeen M, Joshua Z (2017). Occurrence of mycotoxigenic fungi in poultry feeds at live-bird markets. Sokoto J Vet Sci.

[5] Ezekiel CN, Atehnkeng J, Odebode AC, Bandyopadhyay R (2014). Distribution of aflatoxigenic *Aspergillus* section Flavi in commercial poultry feed in Nigeria. Int J Food Microbiol.

[6] Gachomo EW, Mutitu EW, Kotchoni OS (2004). Diversity of fungal species associated with peanuts in storage and the levels of aflatoxins in infected samples. Int J Agric Biol.

[7] Oliveira MS, Rocha A, Sulyok M, Krska R, Mallmann CA (2017). Natural mycotoxin contamination of maize (*Zea mays* L.) in the South region of Brazil. Food Control.

[8] Frimpong GK, Adekunle AA, Ogundipe OT, Solanki MK, Sadhasivam S, Sionov E (2019). Identification and toxigenic potential of fungi isolated from *Capsicum peppers*. Microorganisms.

[9] Sadhasivam S, Britzi M, Zakin V, Kostyukovsky M, Trostanetsky A, Quinn E (2017). Rapid detection and identification of mycotoxigenic fungi and mycotoxins in stored wheat grain. Toxins (Basel).

[10] Gautam AK, Gupta H, Soni Y (2012). Screening of fungi and mycotoxins associated with stored rice grains in himachal pradesh. Int J Theor Appl Sci.

[11] Ekwomadu TI, Gopane RE, Mwanza M (2018). Occurrence of filamentous fungi in maize destined for human consumption in South Africa. Food Sci Nutr.

[12] Reddy KRN, Farhana NI, Salleh B (2011). Occurrence of *Aspergillus* spp. and Aflatoxin B1 in Malaysian Foods Used for Human Consumption. J Food Sci.

[13] Habib MA, Abdu P, Kwanashie CN, Kabir J, Negedu A (2015). Isolation and identification of *Aspergillus* species from poultry feeds in Kaduna State, Nigeria. Microbiol Res Int.

[14] Haruna M, Dangora DB, Khan AU, Batagarawa US, Ibrahim H, Adamu US (2017). Incidence of fungal flora and aflatoxin in some spices sold in Central Market, Funtua, Nigeria. UMYU J Microbiol Res.

[15] Rushing BR, Selim MI (2019). Aflatoxin B1: a review on metabolism, toxicity, occurrence in food, occupational exposure, and detoxification methods. Food Chem Toxicol.

[16] Rossiter S (2019). Aflatoxins in food: overview, meaning and scientific definitions. Int Food Inform Service (IFIS).

[17] David GS, Gary PM (2020). Aflatoxins.

[18] Mahato DK, Lee KE, Kamle M, Devi S, Dewangan KN, Kumar P (2019). Aflatoxins in food and feed: an overview on prevalence, detection and control strategies. Front Microbiol.

[19] Shekhar M, Singh N, Dutta R, Kumar S, Mahajan V (2017). Comparative study of qualitative and quantitative methods to determine toxicity level of *Aspergillus flavus* isolates in maize. PLoS One.

[20] Pradhan S, Ananthanarayan L (2020). Standardization and validation of a high-performance thin-layer chromatography method for the quantification of aflatoxin B1 and its application in surveillance of contamination level in marketed food commodities from the Mumbai region. J Planar Chromatogr - Mod TLC.

[21] Rajeshwari P, Raveesha KA (2016). Mycological analysis and aflatoxin B1 contaminant estimation of herbal drug raw materials. Afr J Tradit Complement Altern Med.

[22] Akhund S, Akram A, Hanif NQ, Qureshi R, Naz F, Nayyar BG (2017). Pre-harvest aflatoxins and *Aspergillus flavus* contamination in variable germplasms of red chillies from Kunri, Pakistan. Mycotoxin Res.

[23] Obonyo MA, Salano EN (2018). Perennial and seasonal contamination of maize by aflatoxins in eastern kenya. Int J Food Contam.

[24] Mohana DC, Thippeswamy S, Abhishek RU, Shobha B, Mamatha MG (2016). Studies on seed-borne mycoflora and aflatoxin B1 contaminations in food based seed samples: molecular detection of mycotoxigenic *Aspergillus flavus *and their management. Int Food Res J.

[25] Mohana DC, Thippeswamy S, Abhishek RU, Shobha B, Mamatha MG (2017). Studies on seed-borne mycoflora and aflatoxin B1 contaminations in food based seed samples: molecular detection of mycotoxigenic *Aspergillus flavus* and their management. Int Food Res J.

[26] Yang XH, Xu GJ, Zhang XY, Li D, Li HX, Sun YF (2017). A fluorescence polarization immunoassay for the detection of aflatoxins in herbal teas. Yaoxue Xuebao.

[27] Ramos CECDO, Damasceno JC, Kazama R, Vieira TSWJ, Zambom MA, Ferreira FG (2016). Seasonal milk contamination by aflatoxin m1, organophosphates and carbamates in Paraná - Brazil. Semin Cienc Agrar.

[28] Siddiqui S, Dharini V, Sarath Chandra G, Periyar Selvam S, Mahesh Kumar M (2020). Determination of aflatoxin and pesticide residues in different Indian chilli varieties. Int J Adv Sci Technol.

[29] Zahra N, Idrees A, Aslam M, Noreen Z, Masood S, Saeed MK (2019). Effect of moisture content on aflatoxin B1 production in wheat flour samples collected from Lahore, Pakistan. Pak J Analyt Environ Chem.

[30] Ajmal M, Akram A, Hanif NQ, Mukhtar T, Arshad M (2021). Mycobiota isolation and aflatoxin B1 contamination in fresh and stored sesame seeds from rainfed and irrigated zones of Punjab, Pakistan. J Food Prot.

[31] Pothiappan P, Vijayakaran K, Sarathchandra G, Gnanaraj PT (2018). Co-occurrence of aflatoxin B1 and Citrinin mycotoxin contaminated feed in piglet diarrhoea. Asian J Microbiol Biotechnol Environ Sci.

[32] Mona EE, Mona MHS, Nagwa SA (2016). Frequency of fungal and aflatoxin B1 contaminants in cattle feed. Int J Pharm Tech Res.

[33] Saleem AR, Al-Johani M (2018). Mycobiota and chromatographic analyses of aflatoxin contamination of *Aspergillus* species isolated from poultry feed. J Food Safety.

[34] Nazir A, Kalim I, Imran M, Bilal MA, Zahra N, Ahmad A (2021). Incidences and bio-detoxification of aflatoxins in rice and cattle feed crops under different agro-ecological zones. Polish J Environ Stud.

[35] Pradeepkiran JA, Matcha B (2018). Analysis of aflatoxin B1 in contaminated feed, media, and serum samples of *Cyprinus carpio* L. by high-performance liquid chromatography. Food Qual Saf.

[36] Starr JM, Rushing BR, Selim MI (2017). Solvent-dependent transformation of aflatoxin B1 in soil. Mycotoxin Res.

[37] Qu LL, Jia Q, Liu C, Wang W, Duan L, Yang G (2018). Thin layer chromatography combined with surface-enhanced raman spectroscopy for rapid sensing aflatoxins. J Chromatogr A.

[38] Ghaemmaghami SS, Pashootan N, Razzaghi-Abyaneh M (2020). Toxigenicity and phylogeny of *Aspergillus* section Flavi in poultry feed in Iran. Curr Med Mycol.

[39] Singh J, Mehta A (2019). Protein-mediated degradation of aflatoxin B1 by *Pseudomonas putida*. Braz J Microbiol.

[40] Al-Salami I (2019). Aflatoxin produced by toxigenic fungi isolated from feedstuff of animal diets. Plant Arch.

[41] Barakat GII, Kamal YN, Sultan AM (2019). Could aflatoxin B1 production by *Aspergillus flavus *affect the severity of keratitis: an experience in two tertiary health care centers, Egypt. Eur J Clin Microbiol Infect Dis.

[42] Akinola SA, Ateba CN, Mwanza M (2019). Polyphasic assessment of aflatoxin production potential in selected aspergilli. Toxins.

[43] Mitema A, Feto NA, Rafudeen MS (2020). Development and validation of TOF/Q-TOF MS/MS, HPLC method and in vitro bio-strategy for aflatoxin mitigation. Food Addit Contam Part A Chem Anal Control Expo Risk Assess.

[44] Moghadam MM, Rezaee S, Mohammadi AH, Zamanizadeh HR, Moradi M (2020). A survey on contamination of Iranian pistachio cultivars to *Aspergillus* section Flavi and Aflatoxin. J Nuts.

[45] Qin L, Li D, Zhao J, Yang G, Wang Y, Yang K (2021). The membrane mucin Msb2 regulates aflatoxin biosynthesis and pathogenicity in fungus *Aspergillus flavus*. Microb Biotechnol.

[46] Bal J, Yun SH, Chun J, Kim BT, Kim DH (2016). Taxonomic characterization, evaluation of toxigenicity, and saccharification capability of *Aspergillus* section Flavi isolates from korean traditional wheat-based fermentation starter nuruk. Mycobiology.

[47] Aiko V, Mehta A (2016). Prevalence of toxigenic fungi in common medicinal herbs and spices in India. 3 Biotech.

[48] David Satcher, ATSDR (1997). A toxicological profile for chloroform.

[49] EPA (2014). Substance profile on chloroform from eleventh report on carcinogens.

[50] ThermoScientific (2011). Application tip of the week: matrix spiking- why spike and how to spike. ThermoScientific application note- Tip log #112.

[51] EuroProxima (2020). Sample spiking and recovery calculation for ELISA tests.

[52] Soleimany F, Jinap S, Rahmani A, Khatib A (2011). Simultaneous detection of 12 mycotoxins in cereals using RP-HPLC-PDA-FLD with PHRED and a post-column derivatization system. Food Addit Contam.

[53] Abbas M, Lukman Bola Abdulra’uf (2021). Chromatographic techniques for estimation of aflatoxins in food commodities. Aflatoxins [Working Title].

[54] Choochuay S, Phakam J, Jala P, Maneeboon T, Tansakul N (2018). Determination of aflatoxin B1 in feedstuffs without clean-up step by high-performance liquid chromatography. Int J Analyt Chem.

[55] ICH (1995). ICH Q 2 (R1) validation of analytical procedures: ICH harmonised tripartite guideline-CPMP/ICH/381/95.

[56] Kamboj A, Saluja AK (2017). Development of validated HPTLC method for quantification of stigmasterol from leaf and stem of Bryophyllum pinnatum. Arab J Chem.

[57] Gurav NP, Medhe S (2018). Analysis of aflatoxins B1, B2, G1 and G2 in peanuts: validation Study. Anal Chem: Indian J.

[58] Bland JM, Altman DG (1999). Measuring agreement in method comparison studies. Stat Methods Med Res.

[59] Zhang K, Banerjee K (2020). A review: sample preparation and chromatographic technologies for detection of aflatoxins in foods. Toxins.

[60] IARC (2012). Chemical and physical characteristics of the principal mycotoxins.

[61] Vaz A, Silva ACC, Rodrigues P, Venâncio A (2020). Detection methods for aflatoxin M1 in dairy products. Microorganisms.

[62] Wacoo AP, Wendiro D, Vuzi PC, Hawumba JF (2014). Methods for detection of aflatoxins in agricultural food crops. J Appl Chem.

[63] Lalah JO, Omwoma S, Orony DAO, Xi-Dai Long (2019). Aflatoxin B1: chemistry, environmental and diet sources and potential exposure in human in Kenya. Aflatoxin B1 occurrence, detection and toxicological effects.

[64] Berthiller F, Crews C, Dall’Asta C, Saeger S De, Haesaert G, Karlovsky P (2013). Masked mycotoxins: a review. Mol Nutr Food Res.

[65] Reiter E, Zentek J, Razzazi E (2009). Review on sample preparation strategies and methods used for the analysis of aflatoxins in food and feed. Mol Nut Food Res.

[66] Capriotti AL, Cavaliere C, Piovesana S, Samperi R, Laganà A (2012). Multiclass screening method based on solvent extraction and liquid chromatography-tandem mass spectrometry for the determination of antimicrobials and mycotoxins in egg. J Chromatogr A.

[67] Salisu B, Marwanis Anua S, Rosli Wan Ishak W, Mazlan N, Lawal U (2020). Incidence, distribution and phenotypic characterisation of aflatoxigenic fungi contaminating commonly consumed food grains in Katsina State, Nigeria. Malays J Med Health Sci.

[68] Salisu B, Anua SM, Rosli Wan IW, Mazlan N (2020). Review on the Aflatoxins’ Contamination of Foods and Public Health Effects among Nigerian Population. UMYU J Microbiol Res.

[69] Albaoukadel K, Kassambara A (2018). Regression model accuracy metrics: R-square, AIC, BIC, Cp and more - articles. Regression model validation.

[70] Salciccioli JD, Crutain Y, Komorowski M, Marshall DC, Leo AC, Peter C, Mohammad MG, Alistair J, Matthieu K, Dominic M (2016). Sensitivity analysis and model validation. Secondary analysis of electronic health records.

[71] Yang N, Philip Wexler (2014). Dichloromethane. Encyclopedia of toxicology.

[72] Chemistry LibreTexts (2019). Thin layer chromatography. Chemistry LibreTexts.

[73] Khayoon WS, Saad B, Yan CB, Hashim NH, Ali ASM, Salleh MI (2010). Determination of aflatoxins in animal feeds by HPLC with multifunctional column clean-up. Food Chem.

[74] Afsah-Hejri L, Jinap S, Arzandeh S, Mirhosseini H (2011). Optimization of HPLC conditions for quantitative analysis of aflatoxins in contaminated peanut. Food Control.

[75] Thomas W, Charle AD (2021). Beer’s law. Molecular and atomic spectroscopy. Chemistry LibreTexts.

[76] Gupta PC (2015). Method’s validation of an analytical procedure. Pharma Tutor.

[77] EC No 401 (2006). Laying down the methods of sampling and analysis for the official control of the levels of mycotoxins in foodstuffs. Off J Eur Union.

[78] Bewick V, Cheek L, Ball J (2003). Statistics review 7: correlation and regression. Crit Care.

[79] Van Egmond HP, Jonker MA, FAO (2004). Mycotoxin regulations in 2003 and current developments. Worldwide regulations for mycotoxins in food and feed in 2003.

[80] Warth B, Ezekiel CNN, Sulyok M, Ezekiel VCC, Babalola DAA, Krska R (2012). Incidence and consumer awareness of toxigenic *Aspergillus* section Flavi and aflatoxin B1 in peanut cake from Nigeria. Food Control.

[81] Kayode OF, Sulyok M, Fapohunda SO, Ezekiel CN, Krska R, Oguntona CRB (2013). Mycotoxins and fungal metabolites in groundnut- and maize-based snacks from Nigeria. Food Addit Contam Part B Surveill.

[82] Adetuniji MC, Atanda OO, Ezekiel CN, Dipeolu AO, Uzochukwu SVA, Oyedepo J (2014). Distribution of mycotoxins and risk assessment of maize consumers in five agro-ecological zones of Nigeria. Eur Food Res Technol.

[83] Okigbo RN, Anene CM (2017). Prevalence of aflatoxin in dried okra (*Abelmoschus esculentus*) and tomatoes (*Lycoperisicon esculentum*) commercialized in Ibadan metropolis. Integr Food Nutr Metab.

[84] Oyedele OA, Ezekiel CN, Sulyok M, Adetunji MC, Warth B, Atanda OO (2017). Mycotoxin risk assessment for consumers of groundnut in domestic markets in Nigeria. Int J Food Microbiol.

[85] Ojuri OT, Ezekiel CN, Sulyok M, Ojuri OT, Ezekiel CN, Sulyok M (2018). Assessing the mycotoxicological risk from consumption of complementary foods by infants and young children in Nigeria. Food Chem Toxicol.

[86] Felagha I (2016). Total aflatoxin contamination of wheat, groundnut and their products sold in three markets within port-harcourt metropolis, Nigeria. J Environ Earth Sci.

[87] Hertveldt K (2016). Mycotoxins occurrence in Nigerian cereal crops (sorghum and millet).

[88] Ogara IM, Zarafi AB, Alabi O, Banwo O, Ezekiel CN, Warth B (2017). Mycotoxin patterns in ear rot infected maize: a comprehensive case study in Nigeria. Food Control.

[89] Ojuri OT, Ezekiel CN, Eskola MK, Šarkanj B, Babalola AD, Sulyok M (2019). Mycotoxin co-exposures in infants and young children consuming household- and industrially-processed complementary foods in Nigeria and risk management advice. Food Control.

[90] Adetunji MC, Alika OP, Awa NP, Atanda OO, Mwanza M (2018). Microbiological quality and risk assessment for aflatoxins in groundnuts and roasted cashew nuts meant for human consumption. J Toxicol.

[91] Akinmusire OO, El-Yuguda AD, Musa JA, Oyedele OA, Sulyok M, Somorin YM (2018). Mycotoxins in poultry feed and feed ingredients in Nigeria. Mycotoxin Res.

[92] Omeiza GKK, Kabir J, Kwaga JKPKPP, Kwanashie CNN, Mwanza M, Ngoma L (2018). A risk assessment study of the occurrence and distribution of aflatoxigenic *Aspergillus flavus* and aflatoxin B1 in dairy cattle feeds in a central northern state, Nigeria. Toxicol Rep.

[93] Momodu O, Adegboyega O, Chibundu NE, Rui K, Patrick B N, Michael S (2016). Detection of *Aspergillus* section Flavi and aflatoxin in locally formulated fish feeds from South-Western Nigeria. J Aquac Res Dev.

